# Editorial

**Published:** 1841-02-20

**Authors:** 


THE MEDICAL EXAMINER.

PHILADELPHIA, FEBRUARY 20, 1841.

### FUNCTIONS OF DIFFERENT PORTIONS OF THE SPINAL MARROW.

THE last number of the Bulletin of the French Academy, contains a detailed case of remarkable injury of the cervical portion of the spinal marrow. Its physiological importance has induced us to translate it for the benefit of our readers. The symptoms following the injury were marked, and free from complication. They consisted in entire loss of motion of the injured side, without alteration of sensation. The autopsy was minute, and exhibited the exact lesions upon which these phenomena depended, viz., a section of the right anterior column of the medulla spinalis. Vivisection of animals, undertaken with a view to determine the functions of different portions of the spinal marrow, must, from their very nature, give imperfect results. Accidents in the case cited, produced the very lesion which science demanded, and which art would have attempted in vain—a complete and isolated section of one of the anterior chords of the medulla. The lesion occurred also in a point most favourable to the exactness of the conclusions deducible from it. Existing opposite the sixth vertebra, some of the origins of the brachial plexus were left above it, and the filaments from these continued to act, while all motion below the injury was completely abolished. The wound was inflicted by a strong and pointed dirk knife, and unaccompanied by pain, tumefaction, heat, or swelling—the section of the medulla was, in a word, uncomplicated. Such a case, occurring in an intelligent man capable of revealing his sensations, is of great value. If it does not entirely remove uncertainty, it serves most strongly to corroborate the opinion now generally entertained, that the anterior chords of the medulla are productive of motion, while sensation is dependant exclusively upon the posterior chords.

The case condensed, will be found under our foreign head. We would have been pleased to contrast with the above an observation, also published in a late number of the Bulletin, reported by Dr. James, of entire loss of *sensation*, without paralysis of *motion*—but we are compelled to crowd so much interesting foreign matter into the present number, in order to leave space in the succeeding ones for several valuable and elaborate original papers, that we reluctantly postpone it.

